# Blood Pressure Increase during Oxygen Supplementation in Chronic Kidney Disease Patients Is Mediated by Vasoconstriction Independent of Baroreflex Function

**DOI:** 10.3389/fphys.2017.00186

**Published:** 2017-03-30

**Authors:** René van der Bel, Müşerref Çalişkan, Robert A. van Hulst, Johannes J. van Lieshout, Erik S. G. Stroes, C. T. Paul Krediet

**Affiliations:** ^1^Department of Internal Medicine, Academic Medical Center at the University of AmsterdamAmsterdam, Netherlands; ^2^Department of Hyperbaric Medicine, Academic Medical Center at the University of AmsterdamAmsterdam, Netherlands; ^3^MRC-Arthritis Research UK Centre of Musculoskeletal Ageing Research, School of Life Sciences, Medical School, University of Nottingham, Queen's Medical CentreNottingham, UK

**Keywords:** chronic kidney disease, hypertension, hyperbaric oxygen supplementation, renal hypoxia, systemic vascular resistance, cardiac output

## Abstract

Renal hypoxia is thought to be an important pathophysiological factor in the progression of chronic kidney disease (CKD) and the associated hypertension. In a previous study among CKD patients, supplementation with 100% oxygen reduced sympathetic nerve activity (SNA) and lowered blood pressure (BP). We aimed to assess the underlying haemodynamic modulation and hypothesized a decreased systemic vascular resistance (SVR). To that end, 19 CKD patients were studied during 15-min intervals of increasing partial oxygen pressure (ppO_2_) from room air (0.21 ATA) to 1.0 ATA and further up to 2.4 ATA, while continuously measuring finger arterial blood pressure (Finapres). Off-line, we derived indexes of SVR, cardiac output (CO) and baroreflex sensitivity from the continuous BP recordings (Modelflow). During oxygen supplementation, systolic, and diastolic BP both increased dose-dependently from 128 ± 24 and 72 ± 19 mmHg respectively at baseline to 141 ± 23 (*p* < 0.001) and 80 ± 21 mmHg (*p* < 0.001) at 1.0 ATA oxygen. Comparing baseline and 1.0 ATA oxygen, SVR increased from 1440 ± 546 to 1745 ± 710 dyn·s/cm^5^ (*p* = 0.009), heart rate decreased from 60 ± 8 to 58 ± 6 bpm (*p* < 0.001) and CO from 5.0 ± 1.3 to 4.6 ± 1.1 L/min (*p* = 0.02). Baroreflex sensitivity remained unchanged (13 ± 13 to 15 ± 12 ms/mmHg). These blood pressure effects were absent in a negative control group of eight young healthy subjects. We conclude that oxygen supplementation in CKD patients causes a non-baroreflex mediated increased in SVR and blood pressure.

## Introduction

Hypertension is a hallmark of chronic kidney disease (CKD). There is substantial evidence that this can be attributed to increased sympathetic nerve activity (SNA) (Converse et al., 1992; Koomans et al., 2004; Neumann et al., 2004; Herzog et al., 2008). The mechanisms underlying increased SNA in CKD are not completely understood. Several studies have reported an attenuation of SNA and blood pressure (BP) following bilateral nephrectomy (Medina et al., 1972; Getts et al., 2006; Gawish et al., 2010). This has founded the concept that the trigger of the enhanced central sympathetic outflow in CKD patients resides in the affected kidneys themself. Deterioration of renal oxygenation by altered renal perfusion and increased metabolic demand has been postulated as a common factor in the progression of CKD (Eckardt et al., 2005; Evans et al., 2013) and nephrogenic sympathetic hyperactivity and hypertension (Converse et al., 1992; Hausberg, 2002; Siddiqi et al., 2009).

In this respect, altered renal chemo-receptor activation in CKD has been studied by various groups (Hausberg and Grassi, 2007; Hering et al., 2007; Park et al., 2008). Of special interest is a study by Hering et al. who exposed CKD patients (mean serum creatinine 5.5 ± 0.3 mg/dL) to 100% oxygen over a non-rebreathing mask for 15 min. This resulted in a 30% reduction in SNA accompanied by a lower pulse pressure (Hering et al., 2007). This response was absent in healthy controls and non-CKD patient populations (Kones, 2011; Stub et al., 2015). Therefore, the observed effects on sympathetic nerve activity and BP were attributed to CKD-specific hypoxia-mediated renal chemo-reflex deactivation. Additional support for the existence of a kidney-derived chemo-reflex, were the observations in non-CKD sympathetically hyperactive patient groups not showing such a response (Ganz et al., 1972; Thomson et al., 2002). Thus, the haemodynamic response to oxygen supplementation appears to be uniquely different in CKD patients.

Ever since, it has been assumed that the underlying mechanism of the BP effects of oxygen supplementation in CKD patients is mediated by a decrease in sympathetic outflow leading to a reduction in systemic vascular resistance (Thukkani and Bhatt, 2013). However, so far this has never been substantiated. Therefore, we set out to revisit and further explore the concept that systemic hyperoxia suppresses vasoconstrictor activity and BP in CKD patients. Our aim was to elaborate on the haemodynamic mechanisms underlying the BP changes as previously reported by others. We hypothesized: (1) that the previously observed decrease in BP is the effect of a decrease in (sympathetically mediated) systemic vascular resistance (SVR), and (2) that this effect is related to the amount of oxygen provided in a dose-dependent fashion.

## Materials and methods

### Participants

We studied 19 CKD patients (14 males, 5 females; age 62 ± 10 years, BMI 25.7 ± 3.7 kg/m^2^, eGFR 23.6 ± 7.2 mL/min/1.73 m^2^). Of all patients, values of hemoglobin and proteinuria were available from clinical routine testing within 3 months before the study. Baseline characteristics, including medication use and disease background are given in Table 1. To verify the known hemodynamic effects of hyperoxia and thereby the accuracy of our methods, we also included a group of eight young healthy subjects (6 males and 2 females, mean age 26 ± 3 years, BMI 23.1 ± 2.7 kg/m^2^). The study was carried out in accordance with the Declaration of Helsinki of the World Medical Association (2013). The Medical Ethics Review Committee of the Academic Medical Center (University of Amsterdam, Amsterdam, The Netherlands) approved the study protocol. Before inclusion all participants provided written informed consent.

**Table 1:** **Baseline characteristics of the CKD patients**.

		**Patients**
Age (years)		62 (10)
Gender (m/f)		14/5
Body weight (kg)		77 (13)
BMI (kg/m2)		25.7 (3.7)
Smoking status	Yes/No	4/15
Systolic/diastolic blood pressure (mmHg)		128 (24)/72 (19)
eGFR (mL/min/1.73 m2)		22.5 (5–40)
Haemoglobin (mmol/L)		7.9 (1.3)^*^
Proteinuria (g/L)		0.53 (0.03–2.8)
Renal disease	Vascular	10
	Glomerulonephritis	4
	Tubulo-interstitial	1
	Polycystic disease	3
	Unknown	1
Antihypertensive medication	Alpha blockers	4
	Beta blockers	10
	ACE inhibitors	6
	ARBs	8
	Calcium antagonists	11
	Diuretics	8

*Data are presented as absolute number or mean (SD) or with range in case the outcome measure is skewed. ACE, angiotensin converting enzyme. ARB, Angiotensin II receptor blocker*.

* *Six patients used erythropoietin-analogs*.

### Normobaric challenge

**Figure 1:**
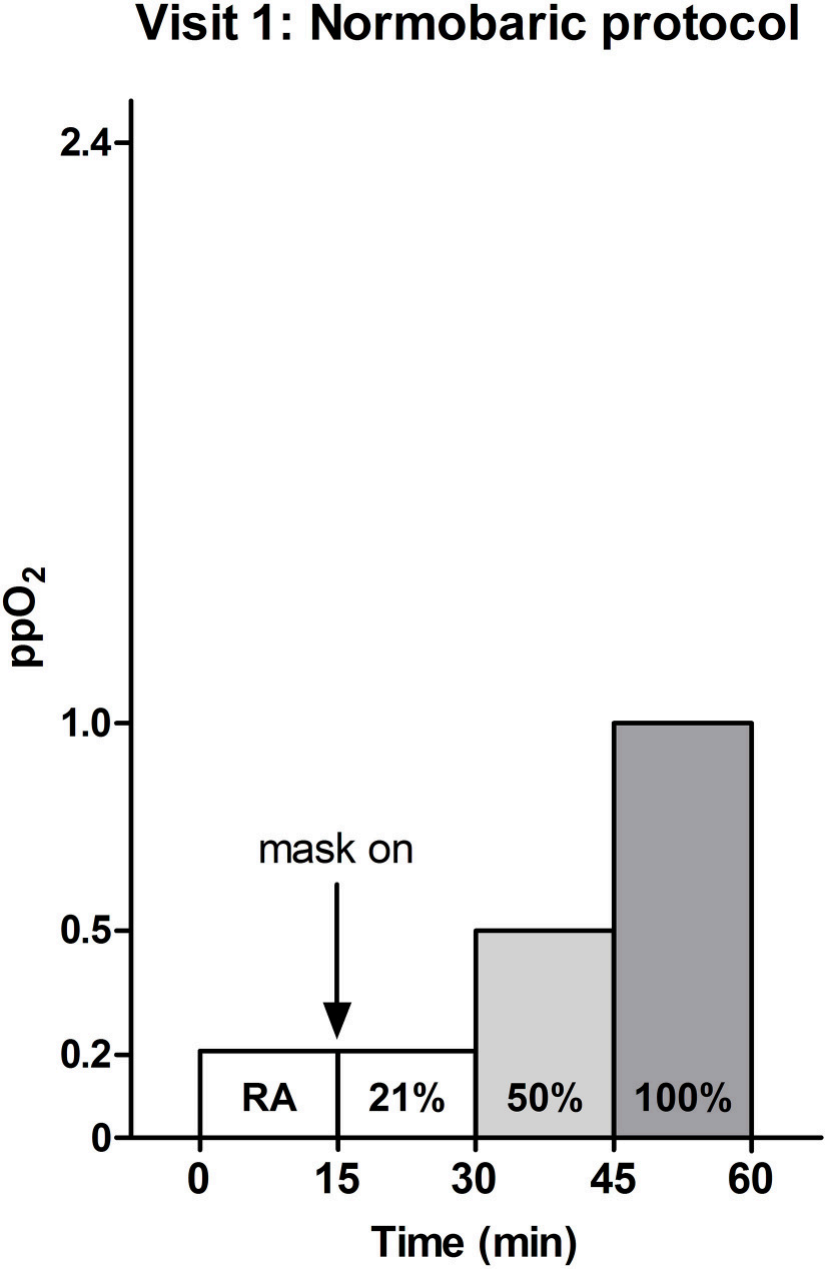
**Normobaric oxygen supplementation protocol**. Room air (RA) and oxygen at different concentrations (21, 50, or 100%) were provided for 15 min each. Measurements were performed at atmospheric pressure, i.e., 1 atmosphere absolute (ATA). At atmospheric pressure a partial oxygen pressure (ppO_2_) of 1.0 ATA is reached, when 100% oxygen is provided.

After an initial baseline measurement of 15 min room air (RA), a non-rebreathing mask was positioned over nose and mouth. Blood pressure measurement (see below) was continued while room air, partial pressure of oxygen (ppO_2_) 0.21 ATA, was provided over the mask at 15 L/min for another 15 min. Thereafter the ppO_2_ in the breathing gas was increased to 50% O_2_ (ppO_2_ 0.5 ATA) and 100% O_2_ (ppO_2_ 1.0 ATA) respectively again for 15 min at each dose (Figure 1). The oxygen dose was regulated using an air-oxygen blender (Precision Medical Inc., Northampton, USA). Patients were blinded to the dosage and were not aware when the oxygen dose was altered. Measurements were performed in a quiet room with the temperature controlled at 22°C. During all measurements, participants remained quietly in the supine position. Patients receiving angiotensin-converting-enzyme (ACE) inhibitors and/or angiotensin II receptor blockers (ARBs) had postponed the intake of these medications until after the study visit.

### Hyperbaric challenge

**Figure 2:**
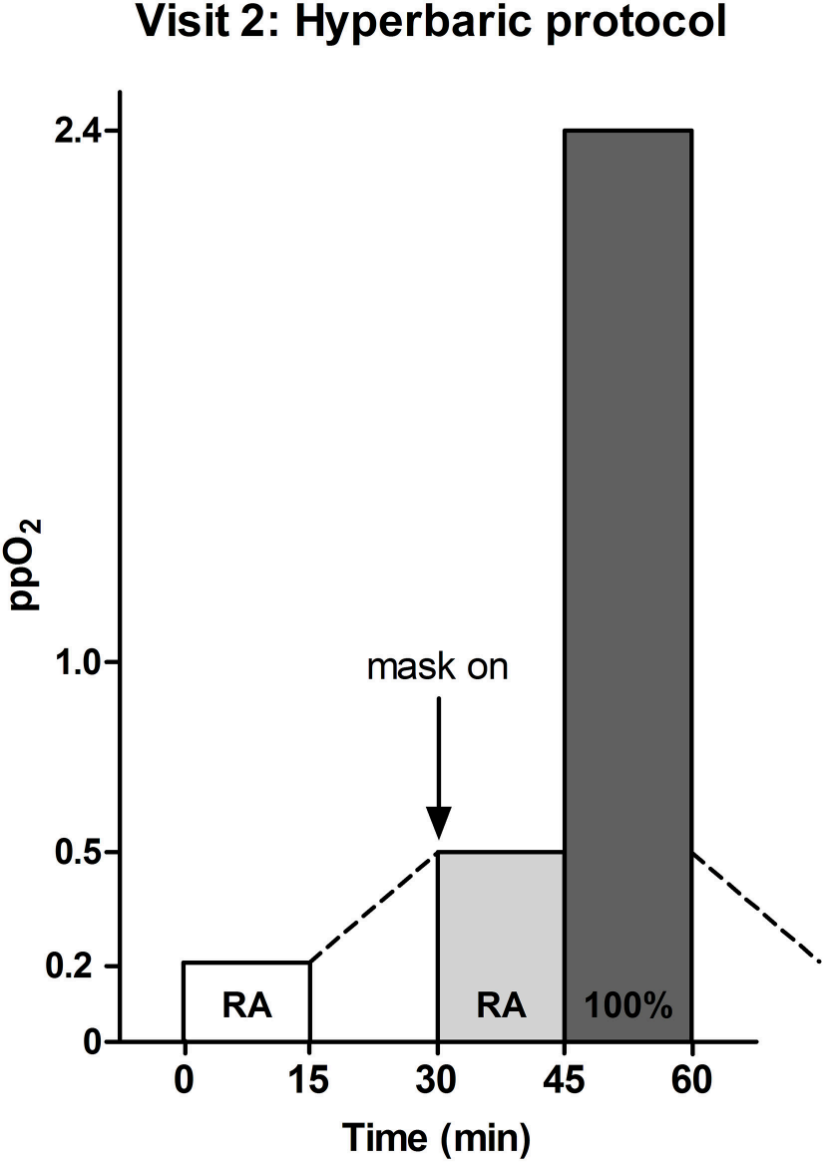
**Hyperbaric oxygen supplementation protocol**. Normobaric and hyperbaric room air (RA) and hyperbaric oxygen (100%) were provided for 15 min each. Measurements were performed at atmospheric pressure (1 ATA) and under hyperbaric conditions (2.4 ATA). At atmospheric pressure a partial oxygen pressure (ppO_2_) of 1.0 ATA was reached, when 100% oxygen was provided. During hyperbaric oxygen supplementation this further increased 2.4-fold, to 2.4 ATA.

In another session, subjects were exposed to hyperbaric oxygen in a hyperbaric chamber. Again, during all measurements patients assumed a supine position. Continuous blood pressure was recorded while breathing room air (RA) at atmospheric pressure (ppO_2_ 0.21 ATA), under hyperbaric conditions at 2.4 ATA (ppO_2_ 0.5 ATA) and at 2.4 ATA during 100% oxygen supplementation (ppO_2_ 2.4 ATA, Figure 2).

### Continuous blood pressure measurements and analysis

During all sessions, continuous blood pressure was measured using finger arterial photo-plethysmography (Portapres™, Finapres Medical Systems, Amsterdam, The Netherlands). The device has been validated for use in CKD patients (Imholz et al., 1998). The appropriate size finger cuff was positioned around the mid-phalanx of the left middle finger for all recordings and passively positioned at heart level. The system had been adapted for use under hyperbaric conditions as previously reported in detail (van der Bel et al., 2016).

The finger arterial pressure signal was recorded at 100 Hz and analyzed off-line using the Modelflow algorithm (Beatscope® version 1.1a, Finapres Medical Systems, Amsterdam, The Netherlands). This algorithm provides a validated beat-to-beat estimate of left ventricular stroke volume (SV), based on a nonlinear 3-element model of the input impedance of the aorta (Jellema et al., 1999). Mean arterial pressure (MAP) was the integral over one heart beat and the heart rate (HR) was the inverse of the pulse interval. Cardiac output (CO) was SV times HR. SVR was MAP divided by CO, in dyn·s/cm^5^. Pulse pressure (PP) was systolic BP (SBP) minus diastolic BP (DBP). All hemodynamic parameters were derived from the last minute of the measurements at baseline and at each oxygen dose. Time domain cross-correlation baroreflex sensitivity (xBRS) was calculated from the same intervals as the other parameters, using dedicated software (WinXBRS 2, BMEye, Amsterdam, The Netherlands; Westerhof et al., 2004). The xBRS was computed using beat-to-beat SBP and R–R interval, in a sliding 10 s window. Each instance that a correlation with a significance level of *p* ≤ 0.01 was found the xBRS value was recorded.

### Statistical analysis

Normal distribution of the data was verified using Levine's test and data are presented as mean ± standard deviation, unless otherwise indicated. The within group responses to increasing ppO_2_ were assessed using general linear modeling. *P* < 0.05 were considered significant.

## Results

### Normobaric oxygen challenge (CKD patients)

**Figure 3:**
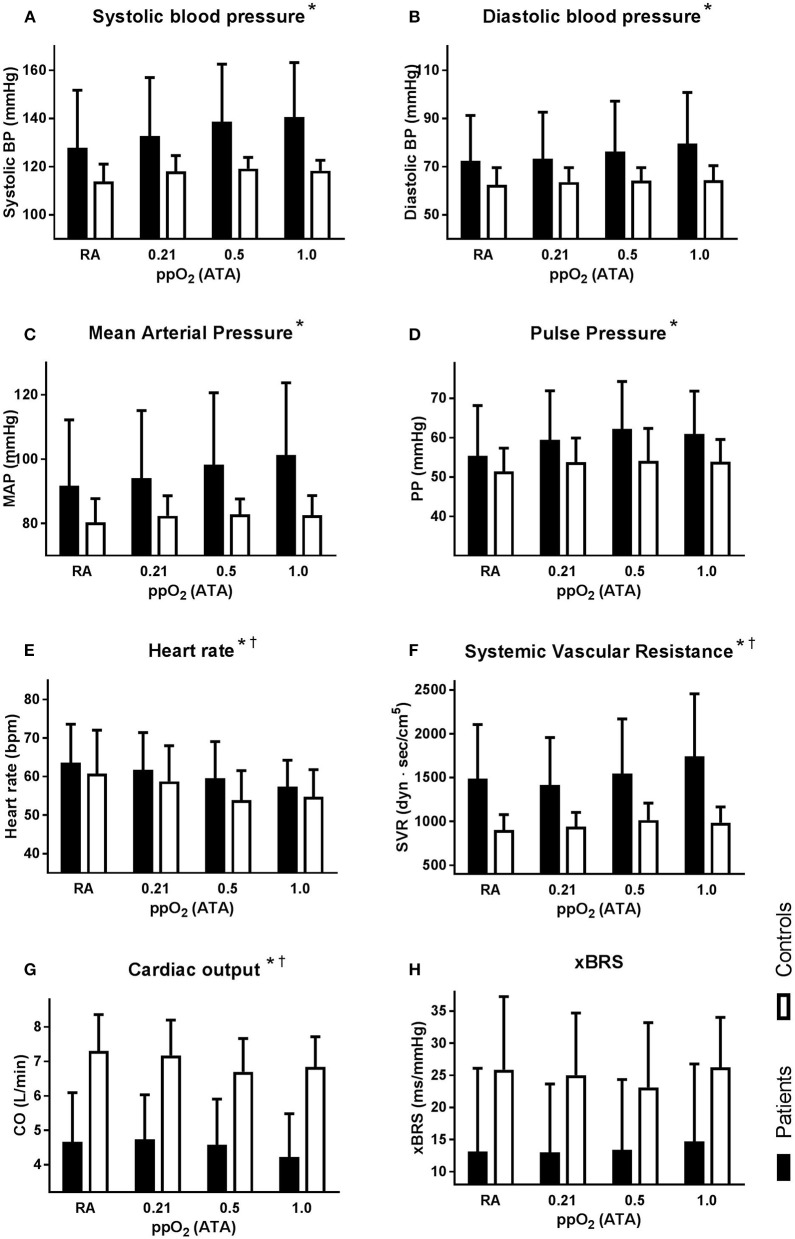
**Hemodynamic response to normobaric oxygen supplementation, for the patient (solid bars) and the young healthy controls (open bars)**. All graphs depict absolute mean ± *SD* at each condition: room air (RA), 21% oxygen over a non-rebreathing mask (ppO_2_ 0.21 ATA), 50% oxygen (ppO_2_ 0.5 ATA), and 100% oxygen (ppO_2_ 1.0 ATA). Averages over the last minute of each condition for: **(A)** systolic blood pressure; **(B)** diastolic blood pressure; **(C)** mean arterial pressure (MAP); **(D)** pulse pressure (PP); **(E)** heart rate (HR); **(F)** systemic vascular resistance (SVR); **(G)** cardiac output (CO); **(H)** baroreflex sensitivity (xBRS). Designation of significant responses to oxygen supplementation in patients ^*^ and in controls^†^.

SBP and DBP both increased with increasing oxygen supplementation from 128 ± 24/72 ± 19 at baseline to 141 ± 23/80 ± 21 mmHg systolic/diastolic at a ppO_2_ of 1.0 ATA, *F*_(3, 18)_ = 12.6, *p* < 0.001 for SBP and *F*_(3, 18)_ = 8.8, *p* < 0.001 for DBP (Figures 3A,B). The pulse pressure increased as well, from 55 ± 13 to 61 ± 11 mmHg [*F*_(3, 18)_ = 5.8, *p* = 0.002, Figure 3D]. HR [60 ± 8 bpm at baseline; 58 ± 6 bpm at 1.0 ATA ppO_2_, *F*_(3, 18)_ = 25.1, *p* < 0.001] and CO [5.0 ± 1.3 L/min at baseline; 4.6 ± 1.1 L/min at 1.0 ATA ppO_2_, *F*_(3, 18)_ = 3.6, *p* = 0.02] decreased during oxygen supplementation (Figures 3E,G). SVR increased from 1440 ± 546 to 1745 ± 710 dyn·s/cm^5^, [*F*_(3, 18)_ = 4.3, *p* = 0.009, Figure 3F]. xBRS remained unchanged with 13 ± 13 ms/mmHg at baseline and 15 ± 12 ms/mmHg at 1.0 ATA ppO_2_ [*F*_(3, 7)_ = 0.647; *p* = 0.59, Figure 3H].

### Hyperbaric oxygen challenge (CKD patients)

**Figure 4:**
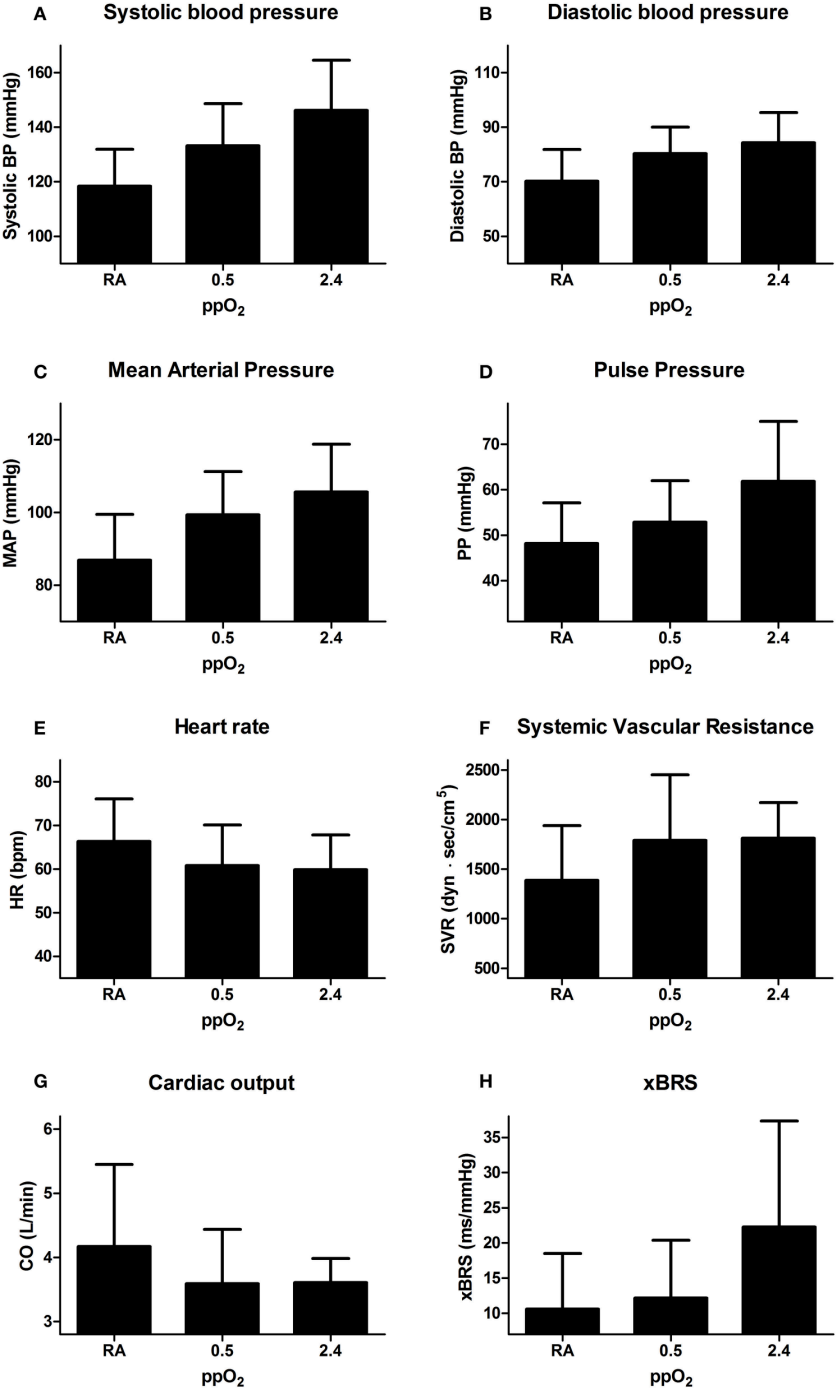
**Hemodynamic response to hyperbaric oxygen supplementation**. All graphs depict absolute mean ± *SD* at each condition: room air (RA), hyperbaric RA (ppO_2_ 0.5 ATA), and hyperbaric oxygen (ppO_2_ 2.4 ATA). Averages over the last minute of blood pressure registration at each condition of: **(A)** systolic blood pressure; **(B)** diastolic blood pressure; **(C)** mean arterial pressure (MAP); **(D)** pulse pressure (PP); **(E)** heart rate (HR); **(F)** systemic vascular resistance (SVR); **(G)** cardiac output (CO); **(H)** baroreflex sensitivity (xBRS).

Due to the results of oxygen supplementation under normobaric conditions, the hyperbaric experiments were suspended for ethical reasons after studying four patients (and not carried out in the control subjects). When changing from a normobaric (1 ATA) to a hyperbaric condition (2.4 ATA, Figure 4), SBP and DBP where 121 ± 17/70 ± 16 at baseline and 146 ± 18/84 ± 11 mmHg systolic/diastolic at a ppO_2_ of 2.4 ATA (Figures 4A,B). Pulse pressure was 51 ± 9 at baseline and 62 ± 13 mmHg at 2.4 ATA ppO_2_ (Figure 4D). HR was 64 ± 9 bpm at baseline and 60 ± 8 bpm at 2.4 ATA ppO_2_ and CO was 4.2 ± 1.3 L/min at baseline and 3.6 ± 0.4 L/min at 2.4 ATA ppO_2_ (Figures 4E,G). No further increase in SVR was observed during hyperbaric oxygen supplementation (Figure 4F). Changes in SBP did not correlate with eGFR (*R* = 0.013).

### Control subjects

During the normobaric oxygen challenge in the control group, SVR increased significantly from 903 at baseline to 985 dyn·s/cm^5^ at a ppO_2_ of 1.0 ATA, *F*_(3, 7)_ = 11.6; *p* < 0.001 (Figure 3F). SBP [*F*_(3, 7)_ = 2.60; *p* = 0.08], DBP [*F*_(3, 7)_ = 1.33; *p* = 0.29], MAP [*F*_(3, 7)_ = 1.28; *p* = 0.31] and PP [*F*_(3, 7)_ = 2.07; *p* = 0.13], did not change (Figures 3A–D), HR [*F*_(3, 7)_ = 13.0; *p* < 0.001] and CO [*F*_(3, 7)_ = 6.73; *p* = 0.002] decreased with oxygen supplementation (Figures 3E,G). xBRS remained unchanged [*F*_(3, 7)_ = 0.884; *p* = 0.47, Figure 3H].

## Discussion

The findings of this study can be summarized as follows: (1) Oxygen supplementation causes a dose-dependent blood pressure increase among CKD patients. (2) This blood pressure increase is caused by an SVR increase. (3) The simultaneous HR decrease with unchanged baroreflex sensitivity indicates that the SVR increase is caused by a direct vascular effect of the increased plasma ppO_2_ rather than a response of the baroreflex.

Our results seem to contradict previous findings in CKD patients (Hering et al., 2007). Hering et al. found that in a similar experiment, exposing CKD patients to 100% oxygen resulted in a 30% reduction in muscle sympathetic nerve activity (Hering et al., 2007), whereas we find an increased systemic vasoconstriction. Upon closer inspection, their SNA decrease was accompanied by a slight increase in diastolic blood pressure—similar to what we found—which was not elaborated upon further. Instead, the analysis focussed on a decreased pulse pressure. However, this rise in diastolic blood pressure may be the key to explaining the decreased SNA during oxygen supplementation in CKD patients. Therefore, we need to consider the haemodynamic effects of hyperoxia in health with regard to baroreflex function.

In healthy humans, oxygen supplementation induces hyperoxic vasoconstriction as observed in our controls and previously reported data (Waring et al., 2003; Gill and Bell, 2004). This response is due to (1) the direct vasoconstrictive effect of plasma pO_2_ itself and (2) its ability to simultaneously hinder vasodilatation by reducing nitric oxide (NO) bioavailability (Waring et al., 2003; Gill and Bell, 2004). In contrast to sympathetically mediated vasoconstriction, hyperoxic vasoconstriction acts independent of baroreflex function (Whalen et al., 1965; Villanucci et al., 1990). CKD patients have an intact arterial baroreflex system (Eckberg and Sleight, 1992), therefore modulation of the baroreflex leads to changes in HR and sympathetic activation to occur simultaneously and in the same direction, i.e., HR increase and sympathetic vasoconstriction versus HR decrease and sympathetic decrease (leading to vasodilation). However, in our experiment vasoconstriction is observed with a simultaneous decrease in HR during oxygen supplementation. This is indicative of a deactivating signal by the baroreflex, resulting in a reduction in HR. Based on the coupling of sympathetic activity and HR, this explains the decrease in sympathetic activity while diastolic blood pressure increases due to direct oxygen driven and non-baroreflex mediated vasoconstriction (Hering et al., 2007).

To explain the blood pressure increase that we observed in CKD patients, we consider the ability of hyperoxia to decrease vasodilatory capacity by reducing NO bioavailability. Reduced NO bioavailability in CKD patients (similar to diabetic and hypertension patients; Al-Waili et al., 2006) may impede the attenuation of the hemodynamic effects of hyperoxic vasoconstriction (Endemann and Schiffrin, 2004; Martens and Edwards, 2011). Therefore, our data are most consistent with inadequate attenuation of hyperoxic vasoconstriction in patients with CKD-related endothelial dysfunction.

Thus, it appears that the hemodynamic response to hyperoxia is not uniquely affected in CKD patients. Instead, it seems that hyperoxic vasoconstriction induces an increase in blood pressure, leading to baroreflex deactivation with a reduction in systemic sympathetic tone. Our data (and in hindsight those from Hering et al.) do not support nor exclude the existence of a CKD-kidney specific hypoxic triggering of (either renal or extra-renal) chemo receptors. The overwhelming effects of oxygen on systemic vasoconstriction render the experimental set-up unsuitable to detect any possible subtle effects of kidney specific oxygenation on sympathetic outflow.

A possible clinical implication of these results is that oxygen supplementation might act as a cardiovascular stressor in CKD patients. Interestingly, this is in line with observations that oxygen supplementation in selected clinical patients is associated with worse outcome (Kones, 2011; Stub et al., 2015). Additionally, our study provides some more explanation on the lack of efficacy of catheter based renal denervation. The presumed decrease in SNA and blood pressure by oxygen supplementation in CKD patients was one of the founding principles of the pathophysiological rationale for renal sympathetic denervation (Schlaich et al., 2011; Davis et al., 2013). Eventually, renal sympathetic denervation showed not to have any effect on blood pressure, and specifically not in CKD patients (Bhatt et al., 2014). Our data question part of the founding rationale for renal sympathetic denervation.

Our study has several methodological limitations that merit discussion. First, patients continued the use of anti-hypertensive medication during the study. For ethical reasons these medications could not be fully withdrawn and was a compromise between taking out possible interfering factors versus patient risk. Our considerations were as follows: because of the specific effects on renal hemodynamics and oxygenation, ACE inhibitors and ARB's were stopped, as other antihypertensive drugs have a less (if any) pronounced effect on RAAS activity or intrarenal oxygen delivery. However, this may only have blunted the hemodynamic effects and thereby would not have affected our eventual conclusions, especially since patients acted as their own control. The same holds for the heterogeneous distributed baseline parameters (e.g., eGFR, smoking status, hemoglobin level) in our relatively small patient group. Secondly, we did not assess changes in CO_2_ partial pressure during oxygen supplementation. However, this has previously been shown not to be influenced by oxygen supplementation (Whalen et al., 1965). Also, the group of young healthy controls was not selected to be age matched, because it intended to verify the accuracy of our method. Others have reported upon the effects of hyperoxia in healthy elderly subjects previously (Whalen et al., 1965; Al-Waili et al., 2006). Lastly, the observers were not blinded but this was corrected by standardizing the time frame selection for analysis.

## Conclusions

We have shown that oxygen supplementation in CKD patients increases blood pressure in a dose dependent fashion. This response is mediated by an increase in SVR, likely as the result of hyperoxic vasoconstriction independent of baroreflex function.

## Author contributions

Study conception and design by RB, RH, ES, CK; Data acquisition, analysis and/or interpretation by RB, MÇ, RH, JL, CK; Drafting of the manuscript by RB, CK; Revising by MÇ, RH, JL, ES, CK; Final approval of manuscript provided by RB, MÇ, RH, JL, ES, CK.

## Funding

This project was funded by the Dutch Kidney Foundation (Project KJPB 12.029 to CP). CP is supported by the Netherlands Organization for Health Research and Development (ZonMw, Clinical Fellowship 40007039712461).

### Conflict of interest statement

The authors declare that the research was conducted in the absence of any commercial or financial relationships that could be construed as a potential conflict of interest.
